# Fully automatic liver segmentation combining multi-dimensional graph cut with shape information in 3D CT images

**DOI:** 10.1038/s41598-018-28787-y

**Published:** 2018-07-16

**Authors:** Xuesong Lu, Qinlan Xie, Yunfei Zha, Defeng Wang

**Affiliations:** 1 College of Biomedical Engineering, South-Central University for Nationalities, Wuhan, 430074 P. R. China; 2 Department of Radiology, Remin Hospital of Wuhan University, Wuhan, 430060 P. R. China; 3 Beijing Advanced Innovation Center for Big Data-Based Precision Medicine, Beihang University, Beijing, China; 4 School of Instrumentation Science and Opto-electronics Engineering, Beihang University, Beijing, China; 5 Research Centre for Medical Image Computing, Department of Imaging and Interventional Radiology, The Chinese University of Hong Kong, Hong Kong, China

## Abstract

Liver segmentation is an essential procedure in computer-assisted surgery, radiotherapy, and volume measurement. It is still a challenging task to extract liver tissue from 3D CT images owing to nearby organs with similar intensities. In this paper, an automatic approach integrating multi-dimensional features into graph cut refinement is developed and validated. Multi-atlas segmentation is utilized to estimate the coarse shape of liver on the target image. The unsigned distance field based on initial shape is then calculated throughout the whole image, which aims at automatic graph construction during refinement procedure. Finally, multi-dimensional features and shape constraints are embedded into graph cut framework. The optimal liver region can be precisely detected with a minimal cost. The proposed technique is evaluated on 40 CT scans, obtained from two public databases Sliver07 and 3Dircadb1. The dataset Sliver07 is considered as the training set for parameter learning. On the dataset 3Dircadb1, the average of volume overlap is up to 94%. The experiment results indicate that the proposed method has ability to reach the desired boundary of liver and has potential value for clinical application.

## Introduction

The extraction of liver tissue is very important for hepatic disease diagnosis, function assessment, and computer-assisted surgery^1^. Among the various medical imaging techniques, computed tomography (CT) is often used for these purposes due to higher signal-to-noise ratio and better spatial resolution. However, it is tedious and time-consuming to get liver regions by manual delineation from several thousand slices. Based on this problem, many researchers have proposed some semi-automatic or automatic methods for liver segmentation^2^. It is interesting to note that most of graph cut methods are still interactive, which need to label the source and sink seeds by operator.

**Figure 1:**
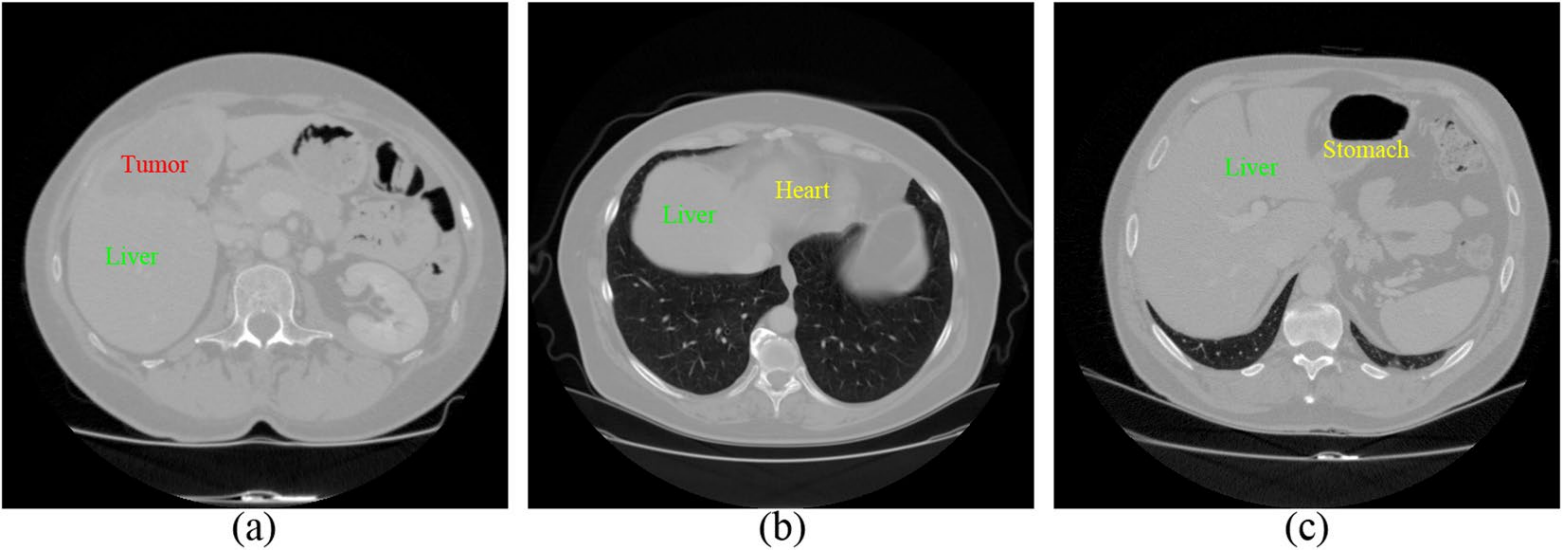
Examples of the limiting factors for liver segmentation in CT images. (**a**) Demonstrates intensity inhomogeneity between liver and tumor. (**b**) and (**c**) display that liver, heart, and stomach share similar intensity value.

However, some factors in CT images bring some challenges to liver segmentation. First, imaging artifacts and tumor pathologies often result in intensity inhomogeneity. Therefore some standard approaches depending on gray-value only, may not be sufficient for this case. Second, intensities of several adjacent organs like heart and stomach are very similar to liver tissue itself. In Fig. 1, some examples of these difficulties are given. Multi-dimensional features and shape priors can aid to separate the neighboring organs with similar intensities and reach the desired boundaries of the structures.

Recently, most studies referenced in a comprehensive review of liver segmentation^3^ focus on three types of methods: deformable model based, level set based and graph cut based methods. To preserve liver shape from the adjacent organs with similar intensities, statistical shape model (SSM)^4^ is often incorporated into these approaches. Chartrand *et al*. presented a semi-automatic approach for liver segmentation^5^. The approximate model of the liver was initialized from a few user-generated contours to globally outline the shape, following by deformation using a Laplacian mesh optimization. Erdt *et al*. combined learned local shape priors with constraints for liver CT segmentation, in order to restrict adaptation to regions with large deformations^6^. Li *et al*. built multiple prior knowledge models to implement liver localization and segmentation^7^. Wang *et al*. proposed a novel adaptive mesh expansion model for liver segmentation^8^. The virtual deformable simplex model was introduced to represent the mesh.

Suzuki *et al*. proposed a two-step automatic method for liver segmentation^9^. The initial localization was achieved using fast marching level set, and then precise refining was finished using geodesic active contour level set. Platero *et al*. developed a variation of level set in which shape priors are incorporated into edge-based and region-based models^10^. Jimenez *et al*. presented an optimal multi-resolution strategy with fine details correction and adaptive curvature, as well as an additional semiautomatic step imposing local curvature constraints for liver surgery^11^. A sparse representation of both global and local image information was embedded in a level set formulation for automated liver segmentation^12^. An automatic algorithm including initial process of a probabilistic atlas with the posteriori classification and following extraction based on level set was developed for liver segmentation^13^.

Graph cut was introduced into segmentation of objects in image data by Boykov *et al*.^14^. An interactive segmentation system was designed for allowing the user to manipulate liver volume by combining graph cut with 3D virtual reality technique^15^. A strategic combination of active appearance model, live wire, and graph cut was proposed to segment the liver^16^. Nakagomi *et al*. presented a novel graph cut algorithm that can take into account multi-shape constraints with neighbor prior constraints^17^. Tomoshige *et al*. employed graph cut based on the shape prior to segment the liver from non-contrast abdominal CT volumes^18^. Shape prior can be estimated through the novel level set based conditional SSM with integrated error model. Li *et al*. proposed a framework consisting of SSM and deformable graph cut for liver segmentation^19^. The mean shape of SSM was moved using thresholding and Euclidean distance transformation to obtain a coarse position in a test image. The final surface of liver was precisely detected by deformable graph cut which can be considered as an optimization process aimed at progressively finding the optimal surface with a minimal cost.

As mentioned above, SSM is helpful to the organ segmentation from complex images. But the construction of SSM is not a trivial task, and heavily relies on the training data. In some cases, large shape and size variabilities from different individuals make it difficult to build a statistical model. In this paper, we aim to automatically and robustly segment livers under graph cut framework without the support of SSM. The initial location of liver in CT images is obtained via transforming the atlas label images. The graph construction is subsequently performed on the unsigned distance field using multi-dimensional features. The desirable region can be extracted by applying the shape constrained graph cut.

## Materials and Methods

**Figure 2:**
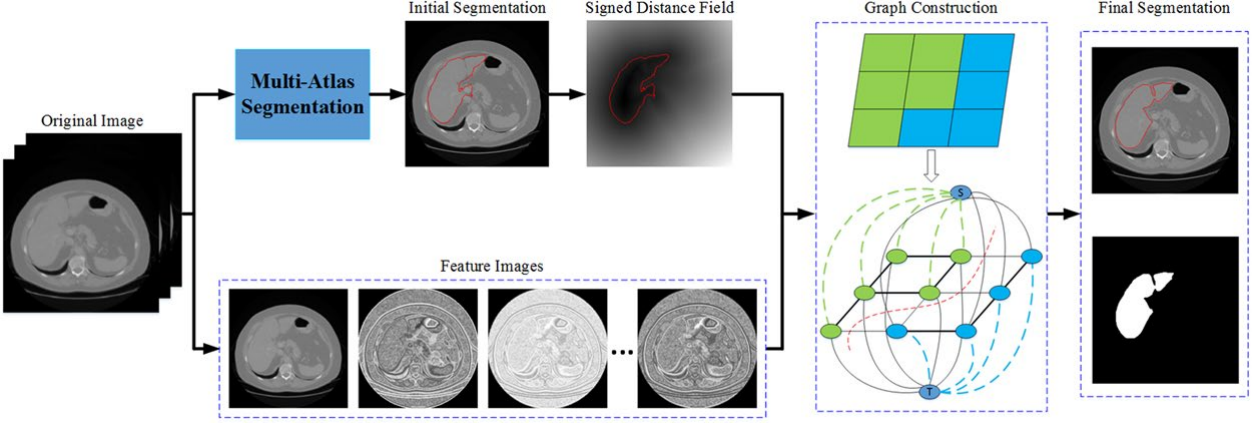
The flowchart of the proposed segmentation framework.

This section describes a coarse-to-fine segmentation framework with no need of user interaction. Figure 2 shows the basic flow of the proposed framework. Firstly, non-rigid registration is performed between the target image to be segmented and atlas intensity image. The initial liver region is detected using atlas label propagation and fusion. Secondly, the unsigned distance field is computed on the whole target image via the initial liver shape. The graph can be constructed on automatic selection of the source and sink seeds. Finally, the original graph cut based on image intensity only is extended by multi-dimensional features and shape constraints. The optimal liver region can be found within a certain range with a minimal cost. It should be noted that the images are treated as 3D manner in all steps rather than 2D slice-by-slice mode.

### Multi-atlas segmentation for initial localization

In order to make our approach automatic, multi-atlas segmentation (MAS) provides a rough delineation for the subsequent procedure. A shape prior can be learned from a representative set of generated contours from atlas images. In general, MAS consists of three important components: registration, atlas selection, and label fusion^20^.

Denote *I* as the target image to be segmented, and denote {(*A*_*i*_, *L*_*i*_)|*i* = 1, …, *N*} as the set of atlases where *A*_*i*_ and *L*_*i*_ are the intensity and label image of the *i*th atlas respectively. For each atlas, MAS generates the warped intensity image *A*′ and corresponding label image *L*′ by an atlas-to-target registration. Here a combined transformation model is employed for the whole registration. An affine model is applied into the global transformation. Then the free-form deformation (FFD) model based on B-splines is^21^ further adopted for the local transformation. To search the optimal results, an adaptive stochastic gradient descent strategy^22^ is chosen for all registrations.

After non-rigid registration, instead of using all warped label images we can make a selection of atlas scans, based on the normalized mutual information (NMI) of *I* and ${A^{\prime} }_{i}$ over the liver structure. It can be formulized as:

1 $${r}_{i}=\frac{NMI(I,{A^{\prime} }_{i};{\rm{\Omega }})}{maxNMI(I,{A^{\prime} }_{j};{\rm{\Omega }})}$$

If it satisfies *r*_*i*_ ≥ *φ*, an atlas *A*_*i*_ should be selected. Hence, a subset of *N*′(*N*′ ≤ *N*) atlases falls into label fusion step. To combine the warped label images of *N*′ atlases into a single segmentation, the weighted version of majority voting^23^ estimates the probability of class *c* at point *p*:

2 $${\rm{L}}(p)=arg\,{\max }_{c\in \{1\cdots k\}}\frac{{\sum }_{i=1}^{N^{\prime} }{\omega }_{i}\cdot \delta [c,{L^{\prime} }_{i}(p)]}{{\sum }_{i=1}^{N^{\prime} }{\omega }_{i}}$$

where c ∈ {1…*k*} is the set of *k* labels; ${\rm{\delta }}[\,\cdot \,]$ is the Kronecker delta function; *ω*_*i*_ = *r*_*i*_ denotes the weight factor which is simply set to accord with the structural similarity in atlas selection stage.

### Automatic graph construction

**Figure 3:**
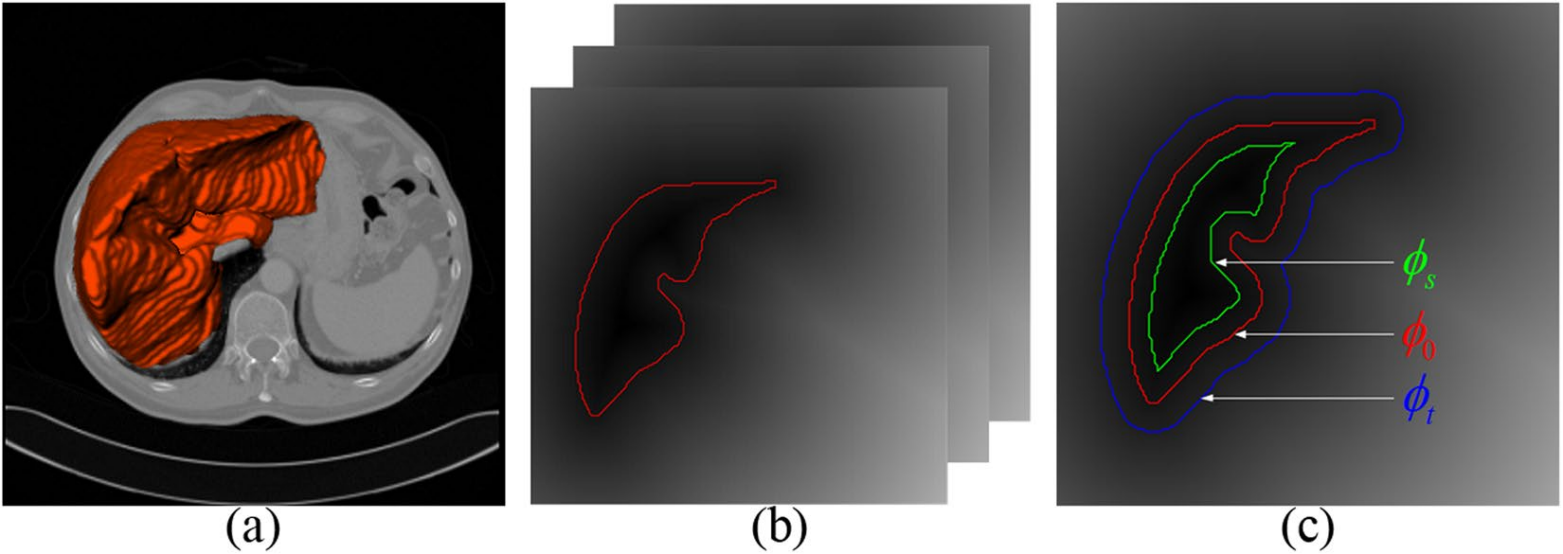
(**a**) The initialized liver shape. (**b**) The unsigned distance field according to the initial segmentation. (**c**) The selection of source and sink seeds on the unsigned distance field.

After the propagated atlas labels are combined using the weighted voting method, an initial region of liver can be obtained. Multi-dimensional graph cut is the critical process in our framework, whose purpose is to precisely extract the liver region based on the initialized liver shape (see Fig. 3a). Subsequently, we build the unsigned distance field according to the initial liver surface. A graph inheriting the initial surface properties is automatically constructed depending on those voxels with zero distance value.

Let *V* be the vertices that are composed of *s* (source), *t* (sink), and the voxels of the target image *I*. Let *E* be the edges that consist of *n*–*links* and *t*–*links*, where *n*–*links* connect the neighboring voxels within the image; *t*–*links* connect the terminal (source or sink) nodes with the voxels of the image. Thus we can construct an undirected *s* − *t* graph G = 〈*V*, *E*〉 for a volumetric data. Unlike the traditional graph cut segmentation in which the source and sink seeds often need to be marked by the users^14^, our graph construction is automated in terms of the unsigned distance field.

As shown in Fig. 3c, denote Φ_0_ as the initial liver surface whose distance value is zero, the source nodes contain the voxels in the interior of Φ_0_ whose distance values are larger than a threshold value Φ_*s*_; the sink nodes contain the voxels in the exterior of Φ_0_ whose distance values are larger than a threshold value Φ_*t*_. Meanwhile, our graph covers the region including the interior of Φ_0_ and the exterior of Φ_0_ whose distance values are smaller than a threshold value Φ_*t*_ rather than the whole image.

### Multi-dimensional graph cut

The multi-dimensional graph cut is driven by cost function derived from the traditional graph cut^14^, which reflects properties of the initial shape. In general, graph cut segmentation can be formulated as a minimization problem of cost function:

3 $${\rm{E}}(L)=\lambda \cdot {E}_{R}(L)+{E}_{B}(L)$$

where *E*_*R*_(*L*) is the regional term, *E*_*B*_(*L*) is the boundary term, and *λ* is the balance coefficient. Based on automatic graph construction above, the proposed shape-constrained cost function is defined as follows:

4 $${\rm{E}}(L)={\sum }_{p\in {\rm{{\rm P}}}}(\alpha \cdot {D}_{p}(L)+\beta \cdot {J}_{p}(L)+\gamma \cdot {S}_{p}(L))+{\sum }_{\{p,q\}\in {\rm{{\rm N}}}}{B}_{p,q}({L}_{p},{L}_{q})$$

where Ρ is a set of pixels with labels L; Ν is a set of all pairs {*p*, *q*} of neighboring elements in Ρ; α, β, and γ are the weight coefficients. The data term *D*_*p*_(*L*), local appearance term *J*_*p*_(*L*), shape term *S*_*p*_(*L*), and boundary term *B*_*p*,*q*_(*L*_*p*_, *L*_*q*_) are defined as follows:

5 $${D}_{p}(L)=\frac{1}{2}\,\mathrm{ln}({(2\pi )}^{d}|\xi |)+\frac{1}{2}{({x}_{p}-\mu )}^{T}{\xi }^{-1}({x}_{p}-\mu )$$

6 $${J}_{p}(L)=\sum _{i=1}^{3}\frac{WD({H}_{p}^{i},{H}_{0}^{i})}{{({\sigma }_{0}^{i})}^{2}}$$

7 $${S}_{p}(L)=1-exp(-\frac{d(p,{{\rm{\Phi }}}_{0})}{{r}_{0}})$$

8 $${B}_{p,q}({L}_{p},{L}_{q})=\exp (-\frac{{\omega }_{pq}\cdot ||{x}_{p},{x}_{q}|{|}^{2}}{2{\sigma }_{1}^{2}})\cdot \frac{1}{dist(p,q)}$$

where μ, ξ are the mean vector and covariance matrix of *d*-dimensional Gaussian model, and *x*_*p*_ is *d*-dimensional features of pixel *p*; ${H}_{p}^{i}$ is the cumulative histogram of the *i*th local binary pattern (LBP) features^24^ at *p* in a local window Ο(*p*), ${H}_{0}^{i}$ is the mean cumulative histogram of the *i*th LBP features on seed regions with variance ${\sigma }_{0}^{i}$, and WD(⋅,⋅) is the L1 Wasserstein distance^25^; d(*p*, Φ_0_) is the distance from *p* to the current shape Φ_0_ (d = 0 when *p* is in the interior of Φ_0_), and *r*_0_ is the radius of a sphere enclosing the current shape; *ω*_*pq*_ is the weight by calculating the Earth Mover’s Distance (EMD)^26^ of two histogram descriptors like the spin images^27^, ||⋅,⋅|| is the Euclidean distance, and *σ*_1_ is the estimated variance.

Given a center voxel *c* in a volume data, VLBP (volume local binary pattern) thresholds the neighboring voxels *p* (p = 0, …, P − 1) within a local region (radius R in XY plane, interval L in Z direction) and generates a binary pattern code as follows:

9 $$VLB{P}_{L,P,R}=\sum _{p=0}^{P-1}s({g}_{p}-{g}_{c}){2}^{p}$$

where *g*_*c*_ and *g*_*p*_ denote the gray value of the center voxel and its neighborhood voxels; s(*x*) is 1 if x ≥ 0 and 0 if x < 0. When the number of neighboring points increases, it is difficult to extend VLBP since that the number of patterns will become very large. We generate simplified descriptors by concatenating local binary patterns on three orthogonal planes (XY, XZ, and YZ), which consider only the co-occurrence statistics in these three directions.

To further address the ambiguous boundary between the liver and adjacent organs, a rotation-invariant and discriminative descriptor is proposed to penalize the boundary term. Given an image patch centered on voxel *p* with radius *r*, each voxel inside the patch is contributed to the 2D histogram along coordinate distance *d* and voxel intensity *i* directions^27^:

10 $$\exp (-\frac{{(d-{d}_{b})}^{2}}{2{\sigma }_{d}^{2}}-\frac{{(i-{i}_{b})}^{2}}{2{\sigma }_{i}^{2}})$$

where *d*_*b*_ and *i*_*b*_ are the histogram bin values in two directions; *σ*_*d*_ and *σ*_*i*_ are the estimated variances. If voxels *p* and *q* are very different, *ω*_*pq*_ in Eq. (8) will be high which will make the energy function smaller. The *x*_*p*_ and *x*_*q*_ in ||*x*_*p*_, *x*_*q*_|| are both 2-dimensional features that include original image and its Gaussian blur image at scale σ = 1.

### Data availability

The raw data used for segmentation to draw the conclusion has been described in section 3. No further material will be provided.

## Experiments and Results

The proposed method was implemented in the Insight Toolkit (ITK). All registrations were performed on the software package *elastix*^28^. The VLBP features and the histogram descriptor for the boundary term were implemented in MATLAB. All programs were run on a 64-bit desktop PC (Intel Dual Core 3.4 GHz CPU and 32 GB Memory).

### Clinical datasets

The two contrast-enhanced CT datasets were adopted for validation. The first public dataset was from Sliver07, which contains 20 CT scans with ground truth. The image resolutions were 512 × 512 × 64~394 voxels. The pixel spacing varied from 0.58 to 0.82 mm, slice thickness from 1 to 3 mm. The second public dataset was from 3Dircadb1, which contains 20 CT scans with ground truth. The image resolutions were 512 × 512 × 74~260 voxels. The pixel spacing varied from 0.57 to 0.87 mm, slice thickness from 1 to 4 mm. The first set was used for selection of the parameters, while the second set was used for comparison of liver segmentation with parameters tuned on the first set.

### Evaluation measures

To quantitatively evaluate the performance of the proposed method, the Dice coefficient was calculated between segmentation by one method (*V*_*seg*_) and the ground truth (*V*_*ref*_):

11 $${\rm{Dice}}=\frac{2|{V}_{seg}{\cap }^{}{V}_{ref}|}{|{V}_{seg}|+|{V}_{ref}|}$$

The bigger the value is, the better the segmentation result. In addition, we employed five volume and surface based measures: volumetric overlap error (VOE), signed relative volume difference (SRVD), average symmetric surface distance (ASD), root mean square symmetric surface distance (RMSD), and maximum symmetric surface distance (MSD), which were presented by MICCAI 2007 challenge^2^ in detail. The smaller the value is, the better the segmentation result.

### Parameter settings

The segmentation parameters were chosen by trial-and-error on the first dataset. We give detailed parameter settings for each step in this section. During initialization using multi-atlas segmentation, a leave-one-out cross validation was performed on each dataset. For each patient selected as a target, 19 other patients served as atlas images, which comprised 19 × 20 registrations. Because of large inter-subject difference on the first dataset, bone tissue visible in image and a mask covering the liver region within 10 mm were used in affine registration stage.

For non-rigid registration, a multi-resolution scheme with three levels was employed. Gaussian smoothing instead of down-sampling was applied with σ = 4.0, 2.0, and 1.0 voxels for *x*, *y*, and *z* directions. A multigrid approach was applied with a spacing of 40, 20, and 10 mm in all directions for the B-splines FFD. The number of random samples was N = 5000, as well as 800 iterations were used. The threshold φ = 0.8 was set to atlas selection.

In graph construction, we set Φ_*s*_ = 25 and Φ_*t*_ = 35. In multi-dimensional graph cut, the weight coefficients α = β = 10, and γ = 80 with 5 iterations. The VLBP parameters were set to L = 2, P = 8, R = 1 and the local window Ο(*p*) was a cube window of 15 × 15 × 7. As for the boundary term, a 4 × 4 histogram descriptor was generated by *σ*_*d*_ = *σ*_*i*_ = 0.5. The estimated variance *σ*_1_ was set to 0.1. The balance coefficient *λ* for interactive graph cut using image intensity only was set to 10.

### Results on the Sliver07 dataset

**Figure 4:**
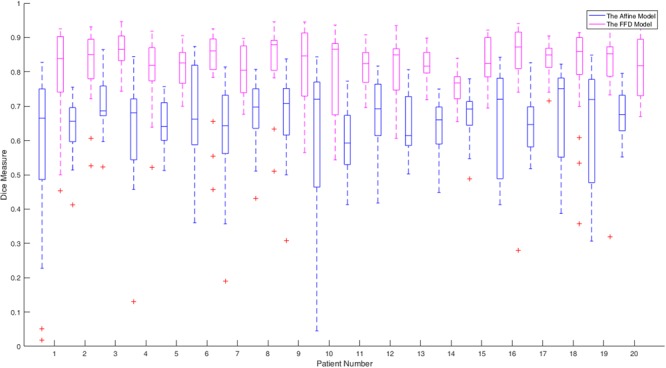
The boxplot of Dice results using the affine and FFD model on the Sliver07 dataset.

After registration, automatic segmentations were generated by warping atlas label images to the target image domain, using the optimal transformation. On the Sliver07 dataset, the boxplot of liver Dice results using the affine and FFD model is shown in Fig. 4 for each patient. It is obvious that registration quality using the FFD model is higher than that of using the affine model, which indicates that the former one could well represent soft tissue deformation. The overall median of liver Dice using the FFD model increases significantly from 0.68 to 0.84, compared with the affine model.

**Figure 5:**
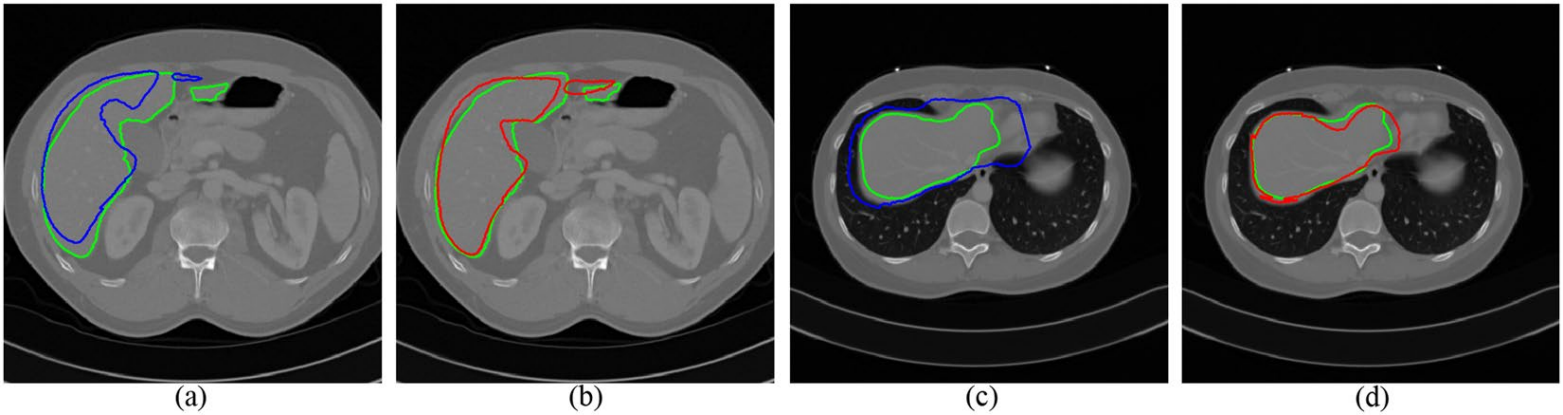
Two examples of segmentation results by different transformation models. The ground truth is shown in green curves. (**a**) and (**c**) Segmentation using the affine model (blue curves). (**b**) and (**d**) Segmentation using the FFD model (red curves).

Figure 5 shows two slices of automatic segmentations by two transformation models. The green curves are the ground truth. The blue curves (see Fig. 5a,c) are the segmentation results using the affine model, while the segmentation results using the FFD model are depicted with red curves (see Fig. 5b,d). It can be seen that the deformed contours through the FFD model are closer to the liver boundary. As the shape initialization of whole framework, a combined segmentation was made by atlas selection and label fusion step.

Table 1 shows the quantitative comparative results of the liver initialization and final segmentation with previous methods^5,29,30^. As shown in the 4th row, the initialization results are the worst in Table 1. Large distance to the ground truth can be seen in the measures of ASD, RMSD, and MSD. Chartrand’s method obtained the best VOE and ASD due to a correction tool for user interaction. Our results show slightly better performance than Chartrand’s method, which RVD, RMSD, and MSD are 1.03%, 1.68 mm, and 12.33 mm respectively.

**Table 1:** The quantitative comparative results for the Sliver07 dataset as mean.

Method	VOE (%)	RVD (%)	ASD (mm)	RMSD (mm)	MSD (mm)
Chartrand^5^	5.14	1.23	1.04	2.14	21.25
Saddi^29^	7.60	3.00	1.30	2.90	24.40
Zheng^30^	7.83	5.06	1.06	1.39	11.12
Initialization	12.71	6.55	4.20	4.43	22.81
Final Result	5.92	1.03	1.06	1.68	12.33

### Results on the 3Dircadb1 dataset

**Figure 6:**
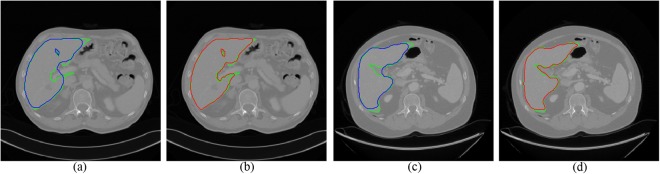
Two examples of segmentation results by different methods. The ground truth is shown in green curves. (**a**) and (**c**) Segmentation using original graph cut (blue curves). (**b**) and (**d**) Segmentation using the proposed method (red curves).

**Figure 7:**
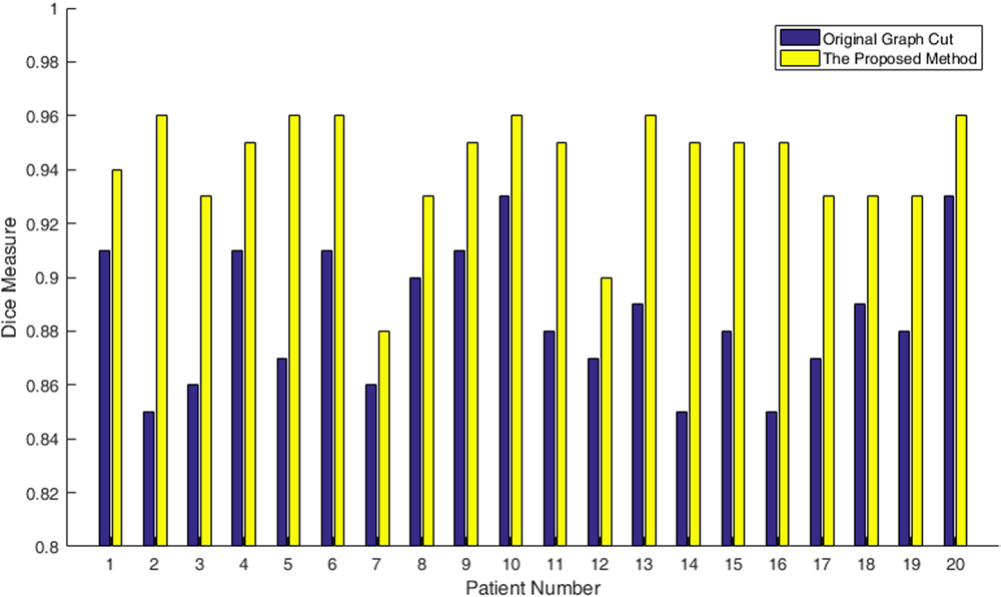
The comparison of Dice measure using two methods on the 3Dircadb1 dataset.

To test on the 3Dircadb1 dataset, we compared the proposed method with interactive graph cut using image intensity only. Figure 6 shows two slices of segmentation results by two methods. The green curves are the ground truth of liver. The blue curves in Fig. 6a,c are the segmentation results using original graph cut. It can be found that some vessels are included. With the help of multi-dimensional graph cut, these vessels can be excluded, as shown in Fig. 6b,d with red curves. The comparison of Dice measure using two methods is shown in Fig. 7. Compared to original graph cut, the mean of Dice measure increases significantly from 0.88 to 0.94.

To evaluate our results more, we also compared the proposed method with several recent methods. Table 2 shows the quantitative comparative results of the liver segmentation with these methods^31–34^. With respect to SRVD, Chung’s method and Kirschner’s method caused under-segmentation of livers. The proposed method achieved much better performance than them except for MSD. Foruzan’s method obtained the best ASD and RMSD due to a generalized profile model. Our results show slightly better performance than Lu’s method whose SRVD and MSD are 0.97 mm and 33.14 mm, respectively.

**Table 2:** The quantitative comparative results for the 3Dircadb1 dataset as mean and standard deviation.

Method	VOE (%)	SRVD (%)	ASD (mm)	RMSD (mm)	MSD (mm)
Chung^31^	12.99 ± 5.04	−5.66 ± 5.59	2.24 ± 1.08	—	25.74 ± 8.85
Kirschner^32^	—	−3.62 ± 5.50	1.94 ± 1.10	4.47 ± 3.30	34.60 ± 17.70
Foruzan^33^	10.39 ± 2.45	1.48 ± 3.59	1.66 ± 0.48	3.68 ± 1.54	35.80 ± 16.00
Lu^34^	9.36 ± 3.34	0.97 ± 3.26	1.89 ± 1.08	4.15 ± 3.16	33.14 ± 16.36
Initialization	16.22 ± 5.11	7.42 ± 6.03	4.03 ± 1.94	7.09 ± 3.53	41.02 ± 16.74
Final Result	9.21 ± 2.64	1.27 ± 3.85	1.75 ± 1.41	3.95 ± 2.26	36.17 ± 15.90

**Figure 8:**
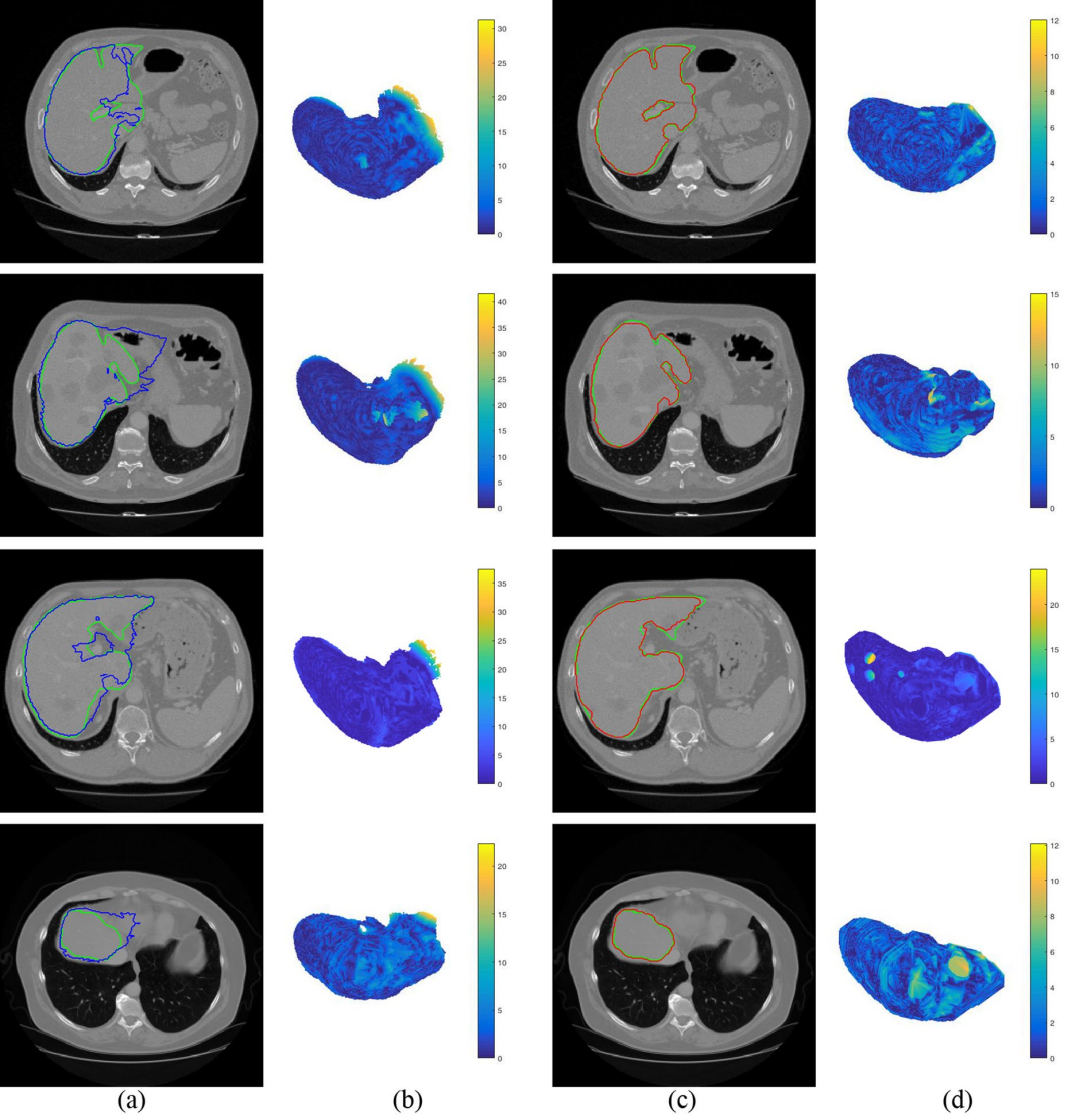
Liver initial and final segmentation results with four difficult cases. The ground truth is shown in green curves. (**a**) Initial segmentation (blue curves). (**b**) Surface distance between initial segmentation to ground truth. (**c**) Final segmentation (red curves). (**d**) Surface distance between final segmentation to ground truth.

As can be seen in the 5th row of Table 2, initial shape of liver is far from final segmentation. Figure 8 shows the initial and final segmentation results with four difficult cases. The first row in Fig. 8a,c show that intensities of stomach and liver are almost same. MAS depending on intensity only can be applied to reach most of the target boundary, except the edges intersecting with two organs. After using multi-dimensional graph cut, the maximal distance to target boundary decreases from 32.32 mm to 12.04 mm (see the first row in Fig. 8b,d). The second row in Fig. 8a,c shows that there are some tumors in this CT image. This difficulty leads to 41.63 mm of the maximal distance to target boundary on initialization stage. After the initial shape is adapted, the maximal distance is decreased to 15.08 mm (see the second row in Fig. 8b,d). The third row in Fig. 8a,c shows that separating the sharp structures and the vessel is challenge. The maximal distance through coarse-to-fine segmentation decreases from 37.48 mm to 26.83 mm (see the third row in Fig. 8b,d). It can be observed from the fourth row of Fig. 8a,c that the boundary between liver and heart is hard to be distinguished. The maximal distance to target boundary is 11.98 mm on final segmentation, as well as that of initialization is 27.28 mm (see the fourth row in Fig. 8b,d).

## Discussions and Conclusions

We have developed a novel approach for automatic liver segmentation, which integrates the initial shape and multi-dimensional graph cut. Our aim is to tackle the problems from the anatomical structure and image quality of liver tissue smartly, without the support of SSM. The initial shape regarded as prior information was caught by means of MAS on atlas images. Multi-dimensional features instead of image intensity only were then embedded into graph cut framework for accurate segmentation. The proposed method was evaluated on 40 CT scan images, which are publicly available. By comparing with original graph cut and recent liver segmentation methods, our method demonstrated effectiveness and veracity for liver detection.

From the results as shown in the last two rows in Table 2, all measures decrease drastically from initialization to final segmentation. Clearly, the step of multi-dimensional graph cut is able to refine the results of MAS. In other words, the final segmentation is affected by initial localization since shape constraint. In this study, common majority voting algorithm was used to learn the shape knowledge from atlas images for liver initialization. However, the VOE, ASD, and RMSD of initialization are 16.22% ± 5.11%, 4.03 ± 1.94 mm, and 7.09 ± 3.53 mm. In the future more sophisticated MAS algorithm^35^ will be applied to initialization step for better shape prior.

Although the overall encouraging results, segmentation accuracy needs to be improved for clinical application. As can be found from Table 2, the MSD of final result is still high to 36.17 ± 15.90 mm. One reason for large surface distance could be the separation of liver and vessels. It is noted that the learned liver shape is incorporated into the regional term of cost function. The boundary term regularized by shape prior^17^ might give a chance to further improve large surface distance. Based on enough automation of the proposed method, extension of multi-shape segmentation with prior knowledge is another subject for future work.
